# Lymphocytic choriomeningitis arenavirus requires cellular COPI and AP-4 complexes for efficient virion production

**DOI:** 10.1128/jvi.02006-23

**Published:** 2024-02-09

**Authors:** Owen Byford, Amelia B. Shaw, Hiu Nam Tse, Eleanor J. A. A. Todd, Beatriz Álvarez-Rodríguez, Roger Hewson, Juan Fontana, John N. Barr

**Affiliations:** 1 School of Molecular and Cellular Biology, Faculty of Biological Sciences, University of Leeds, Leeds, United Kingdom; 2 Astbury Centre for Structural Molecular Biology, University of Leeds, Leeds, United Kingdom; 3 Virology and Pathogenesis Group, National Infection Service, Public Health England, Porton Down, United Kingdom; University Medical Center Freiburg, Freiburg, Germany

**Keywords:** arenavirus, LCMV, COPI, AP-4

## Abstract

Lymphocytic choriomeningitis virus (LCMV) is a bisegmented negative-sense RNA virus classified within the *Arenaviridae* family of the *Bunyavirales* order. LCMV is associated with fatal disease in immunocompromized populations, and as the prototypical arenavirus, acts as a model for the many serious human pathogens within this group. Here, we examined the dependence of LCMV multiplication on cellular trafficking components using a recombinant LCMV expressing enhanced green fluorescent protein in conjunction with a curated siRNA library. The screen revealed a requirement for subunits of both the coat protein 1 (COPI) coatomer and adapter protein 4 (AP-4) complexes. By rescuing a recombinant LCMV harboring a FLAG-tagged glycoprotein (GP-1) envelope spike (rLCMV-GP1-FLAG), we showed infection resulted in marked co-localization of individual COPI and AP-4 components with both LCMV nucleoprotein (NP) and GP-1, consistent with their involvement in viral processes. To further investigate the role of both COPI and AP-4 complexes during LCMV infection, we utilized the ARF-I inhibitor brefeldin A (BFA) that prevents complex formation. Within a single 12-h cycle of virus multiplication, BFA pre-treatment caused no significant change in LCMV-specific RNA synthesis, alongside no significant change in LCMV NP expression, as measured by BFA time-of-addition experiments. In contrast, BFA addition resulted in a significant drop in released virus titers, approaching 50-fold over the same 12-h period, rising to over 600-fold over 24 h. Taken together, these findings suggest COPI and AP-4 complexes are important host cell factors required for the formation and release of infectious LCMV.

**IMPORTANCE:**

Arenaviruses are rodent-borne, segmented, negative-sense RNA viruses, with several members responsible for fatal human disease, with the prototypic member lymphocytic choriomeningitis virus (LCMV) being under-recognised as a pathogen capable of inflicting neurological infections with fatal outcome. A detailed understanding of how arenaviruses subvert host cell processes to complete their multiplication cycle is incomplete. Here, using a combination of gene ablation and pharmacological inhibition techniques, we showed that host cellular COPI and AP-4 complexes, with native roles in cellular vesicular transport, were required for efficient LCMV growth. We further showed these complexes acted on late stages of the multiplication cycle, post-gene expression, with a significant impact on infectious virus egress. Collectively, our findings improve the understanding of arenaviruses host-pathogen interactions and reveal critical cellular trafficking pathways required during infection.

## INTRODUCTION

The *Arenaviridae* family within the *Bunyavirales* order of segmented negative-sense RNA viruses currently compromises 56 species, which are sub-divided into four genera: *Antennavirus*, *Hartmanivirus*, *Mammarenavirus*, and *Reptarenavirus*. Members of the *Mammarenavirus* genus can infect mammals (1–3) and notable species include Lassa virus (LASV) and Junín virus (JUNV), for which infections can result in hemorrhagic fever and subsequent fatality in approximately 1–2% and 30% of human cases, respectively (4, 5). Currently, no specific antiviral therapeutics or FDA-approved vaccines exist to target any member of the *Arenaviridae* family (6). Combined, these factors contribute to the classification of LASV, JUNV, and several other arenaviruses as hazard group 4 pathogens, requiring the highest biosafety level (BSL) 4 containment for their study, which has hindered research progress.

Lymphocytic choriomeningitis virus (LCMV) is classified within the *Mammarenavirus* genus and causes a persistent infection within the common house mouse, *Mus musculus*, which acts as the primary host. Rodent-to-human transmission frequently occurs but rarely results in severe disease (7). LCMV exhibits tropism for non-differentiated neuroblasts and in some populations, including immunocompromized patients or neonates, can lead to aseptic meningitis (8, 9). Specifically, the LCMV Armstrong strain acts as an effective research model for more pathogenic mammarenaviruses due to shared structural and functional characteristics, and requirement for more amenable BSL-2 containment facilities for its study. The LCMV genome comprises small (S) and large (L) segments and encodes four structural proteins using an ambi-sense transcription strategy. Following entry, the input virion-associated (vRNA) S and L segments are transcribed by the viral RNA-dependent RNA polymerase (RdRp) to generate mRNAs encoding nucleocapsid protein (NP) and RdRp, respectively. Subsequent vRNA replication yields S and L anti-genome (ag) RNAs, which act as templates for the transcription of mRNAs encoding a glycoprotein precursor (GPC) and matrix protein (Z) (10, 11). Post-translational cleavage of GPC results in the production of N-terminal glycoprotein-1 (GP-1), C-terminal glycoprotein-2 (GP-2), and stable signal peptide (SSP) that remain associated as trimers (12–15).

Although LCMV has been studied heavily from an immunological perspective, many molecular details underpinning its multiplication are yet to be determined. It is thought that LCMV first attaches to the cell at the plasma membrane (PM) by binding to cellular receptor α-dystroglycan, although members of the Tyro3, Axl, and Mer (TAM) family and C-type lectins have also been implicated (16, 17). LCMV is internalized, potentially via macropinocytosis due to the requirement of actin remodeling and Pak1 (18), after which virions are trafficked via multivesicular bodies (MVBs) to late endosomes. Here, following interaction with secondary receptor CD164, GP-2 mediates endosomal fusion and subsequent release of the vRNAs into the cytosol (19, 20). The site of arenavirus RNA synthesis is unclear, with reports of newly made mRNAs located within processing bodies (21) and agRNA and vRNAs compartmentalized within specialized NP-induced organelles known as replication-transcription complexes (22). Following translation, GPC is glycosylated within the endoplasmic reticulum (ER) then post-translationally cleaved by the resident ER signal peptidase to release the SSP, which remains associated with the GPC while it is trafficked to, and throughout, the Golgi (23). Here, GPC is further cleaved by subtilisin kexin isozyme-1/site-1 protease (SKI-1/S1P) to release GP-1 and GP-2, which associate alongside SSP as trimers (24–26). While the site of viral budding is unclear, virion assembly requires Z-mediated recruitment of endosomal sorting complex required for transport (ESCRT) components; Z is proposed to recruit RNPs via interaction with NP, and myristylation of Z is required for virion incorporation of glycoprotein trimers (27, 28).

Here, using a curated siRNA library targeting the expression of cellular proteins involved in vesicular trafficking, we showed the efficient LCMV multiplication in the neuronal cell line SH-SY5Y required components of the coat protein I (COPI) and adaptor protein 4 (AP-4) complexes. Quantification of LCMV-specific vRNA, protein expression, and viral titers during single-step growth, alongside brefeldin A (BFA) time-of-addition assays, indicated COPI and AP-4 complexes are not required for efficient gene expression, but are important for LCMV assembly and egress. Overall, this study improves our understanding of arenavirus-host interactions, which may aid in the identification of targets for effective anti-arenaviral therapies.

## RESULTS

### Identification of the trafficking components required during LCMV multiplication

**Fig 1:**
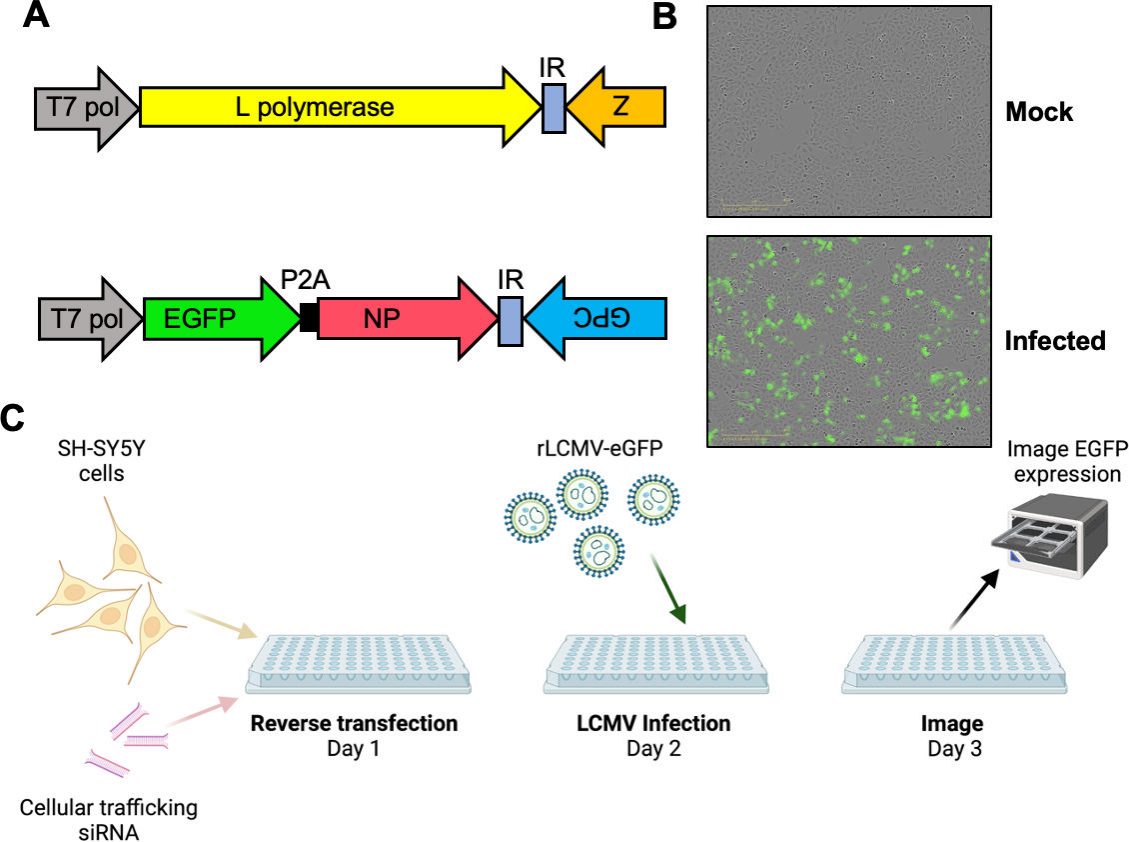
Rescue of a recombinant LCMV expressing eGFP used to perform a siRNA screen to identify cellular factors involved in LCMV multiplication. (**A**) Schematic of rLCMV segments, showing the location of the eGFP open reading frame within the S segment. (**B**) Live-cell images of A549 cells infected with LCMV-eGFP showing eGFP fluorescence at 24 hpi. (**C**) Schematic of the siRNA screen protocol, created with BioRender.com.

The role of host cell trafficking components in LCMV multiplication and egress was assessed using a focused siRNA library comprising three different siRNAs specific for each of 87 host trafficking genes. To facilitate the screen, a recombinant LCMV expressing eGFP (rLCMV-eGFP) using a P2A-linked (29–32) eGFP open reading frame (ORF) appended to the NP ORF (Fig. 1A) was used, rescued as previously described (32). Using this virus, measurement of the total integrated intensity of eGFP expression (TIIE) represented a surrogate for LCMV multiplication and infection progression (Fig. 1B). Following reverse transfection of each individual siRNA, human-origin neuronal SH-SY5Y cells were infected with rLCMV-eGFP at a multiplicity of infection (MOI) of 0.2 and live-cell images were acquired to measure TIIE at 24 hpi (Fig. 1C). At this time point, rLCMV-eGFP has undergone multiple infection cycles in these cells (32); thus, a significant reduction in TIIE relative to the positive control (virus plus transfection reagent only) could represent inhibition of any stage of the LCMV replication cycle encompassing binding, entry, replication, assembly, or egress.

**Fig 2:**
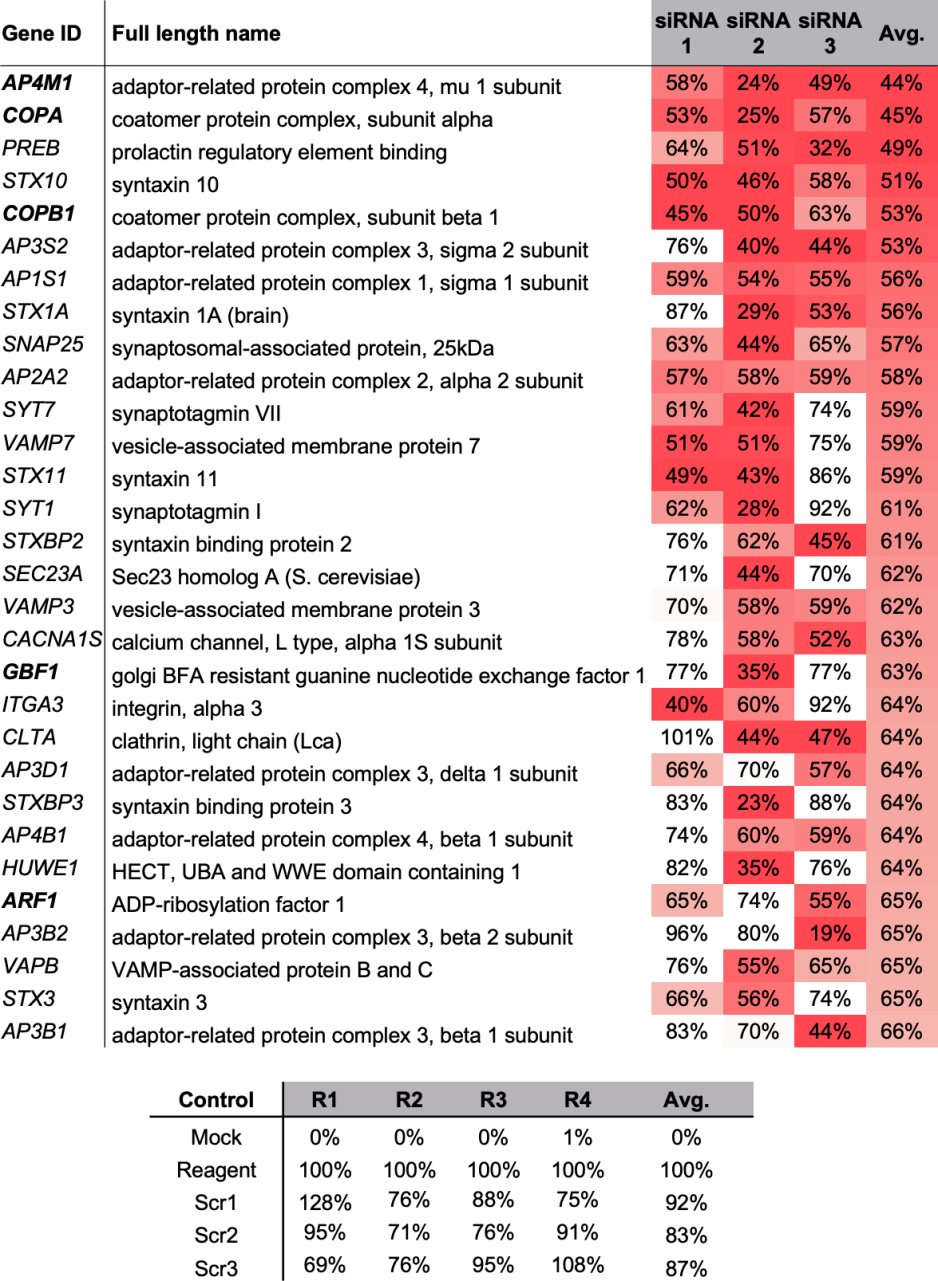
Top 30 gene targets impacting rLCMV-eGFP growth, identified using a siRNA screen of cellular factors involved in vesicle trafficking. Viral growth was measured by total integrated intensity of eGFP expression (TIIE) relative to virus plus transfection reagent control, following siRNA knockdown at 24 hpi. Percentage TIIE values are shown for three individual siRNAs against a single-gene target. Each percentage value represents the mean of four experimental repeats, and the colors indicate the levels of reduction in TIIE from red (most reduction) to white (least reduction). Genes highlighted in boldface represent those associated with COPI and AP-4 vesicles. All transfections included scrambled (scr), mock-infected and infected plus transfection reagent controls, from four repeats, tabulated below.

The impact of each individual siRNA on overall multiplication and infectious virion production was tested four times (supplementary data set S1) and 30 genes with the greatest mean TIIE reduction across all three individual siRNAs are listed in Fig. 2, alongside appropriate controls.

### AP-4 and COPI vesicle components are required for productive LCMV infection

rLCMV-eGFP-mediated TIIE in siRNA transfected cells was most-reduced following knockdown of AP-4 component AP4M1, with a mean TIIE level at 24 hpi of 44% compared to scrambled [Fig. 2; siRNA 1 = 0.5771 (*P* = 0.0091); siRNA 2 = 0.2412 (*P* = 0.0041); siRNA 3 = 0.4935 (*P* = 0.0013)]. In addition, knockdown of a second AP-4 component, AP4B1, resulted in a mean TIIE level of 64%. The second most-reduced TIIE level was for COPI vesicle component COPA, with 45% TIIE compared to scrambled [Fig. 2; siRNA 1 = 0.5335 (*P* = 0.0006); siRNA 2 = 0.2524 (*P* = 0.0013); siRNA 3 = 0.5664 (*P* = 0.0029)], with these siRNAs proving effective for knockdown of the corresponding COPA target and also infection of the related Hazara nairovirus (33). In addition, knockdown of a second COPI component, COPB1, resulted in 53% TIIE.

Interestingly siRNA knockdown of other components required indirectly to form both AP-4 and COPI vesicles, namely the small GTP-binding protein ADP-ribosylation factor 1 (ARF-1) and Golgi BFA-resistant guanine nucleotide exchange factor 1 (GBF-1) (30), also resulted in significantly reduced TIIE levels [Fig. 2; ARF-1; siRNA 1 = 0.6543 (*P* = 0.0185); siRNA 2 = 0.7418 (*P* = 0.0600); siRNA 3 = 0.5500 (*P* = 0.0002) GBF-1; siRNA 1 = 0.7750 (*P* = 0.1478); siRNA 2 = 0.3474 (*P* = 0.0001); siRNA 3 = 0.77 (*P* = 0.0100)]. GBF-1 is a guanine exchange factor (GEF) responsible for activation of ARF-1, which then binds GTP to allow recruitment of intact COPI heptameric complexes and their subsequent anchoring within membranes (34). The recruitment of AP-4 vesicles to the TGN is also regulated by ARF-1, a process mediated by direct interaction between AP4M1 and ARF-1, following its activation (35). Taken together, these findings suggested important roles for both AP-4 and COPI complexes in the LCMV multiplication cycle.

### Generation of a recombinant infectious LCMV bearing a FLAG-tagged GP-1

AP-4 and COPI complexes localize within the Golgi (35–38) and given that LCMV GPC is known to be proteolytically-cleaved and post-translationally modified within this compartment (26), we wished to develop a tool to simultaneously assess GP distribution alongside NP during virus infection.

**Fig 3:**
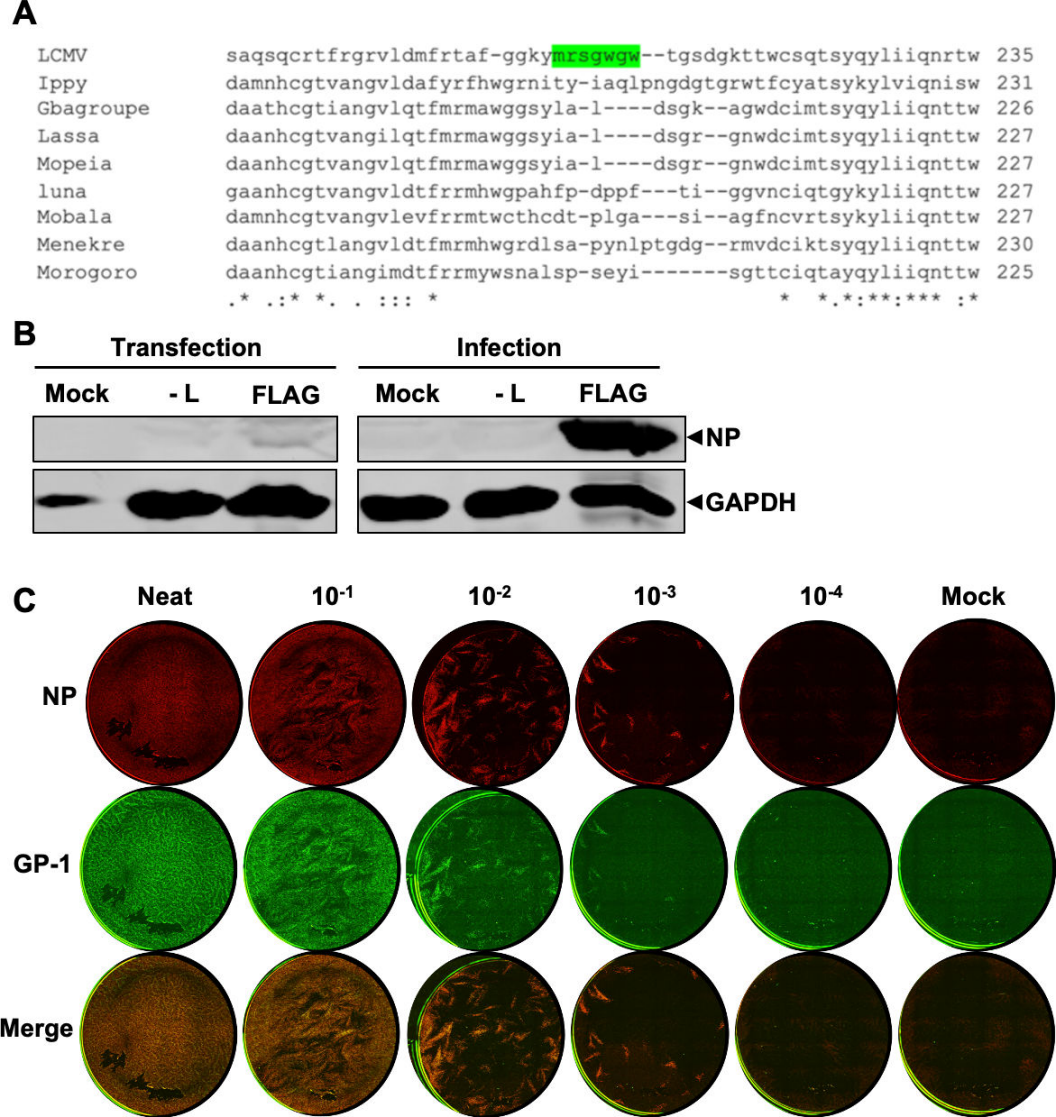
Generation of a recombinant LCMV expressing a FLAG-tagged GP-1. (**A**) Alignment of partial GP-1 sequence of selected Old-World arenaviruses identifying a region of low conservation as a potential FLAG-tag insertion site (green). (**B**) Western blot analysis of transfected BSR-T7 and infected BHK-21 cell cultures confirming LCMV-GP1-FLAG rescue, using antisera specific for LCMV NP and GAPDH as loading control. (**C**) Immunofluorescence focus forming assay for rLCMV-GP1-FLAG showing foci by staining at 3 dpi for LCMV NP, GP-1 (FLAG) alongside merged channels.

To achieve this, we generated rLCMV with a FLAG epitope tag inserted within GP-1 (rLCMV-GP1-FLAG). The site for FLAG insertion was rationally selected by identification of regions of high sequence variation between various OW arenavirus members (Fig. 3A) and a SWISS-MODEL prediction of the GP-1 structure, which revealed the selected site was within a flexible and surface exposed region, thus a suitable candidate for detection by immunofluorescence (IF). Subsequent virus rescue was confirmed by western blotting (Fig. 3B) and using an immunofluorescent focus forming assay (FFA) utilizing NP and FLAG (GP-1) antibody staining, which revealed the resulting virus rLCMV-GP1-FLAG was infectious, the FLAG epitope was confirmed as accessible to antibody detection and yielded viral titers of around 10^5^ pfu/mL (Fig. 3C).

### Analysis of LCMV NP and GP-1 localization

**Fig 4:**
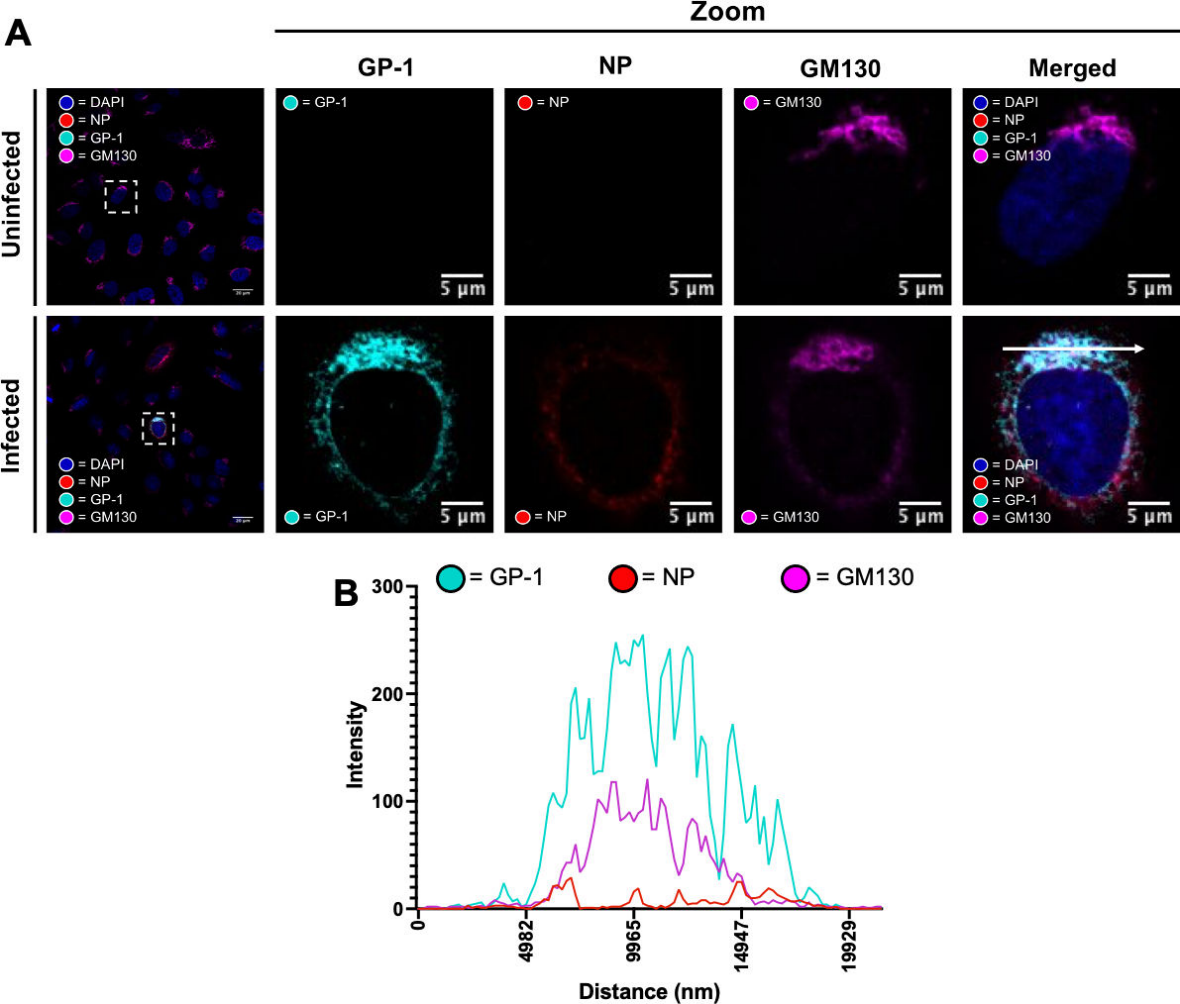
LCMV GP-1 co-localizes with Golgi marker GM130 in rLCMV-GP1-FLAG-infected cells. (**A**) Confocal microscopy of uninfected and rLCMV-GP1-FLAG-infected cells at an MOI of 0.1 at 24 hpi. A549 cells were stained with antisera specific for GM130 (magenta), GP-1 (cyan), and NP (red) alongside DAPI. A zoomed image for both uninfected and infected (white boxes) for all channels is shown, alongside merged channels. (**B**) Line scan for the region highlighted in the zoomed merged image in panel (A) (scan line represented by a white arrow), showing intensities of channels corresponding to NP, GP-1, and GM130 across a ~20 µm distance.

IF analysis of rLCMV-GP1-FLAG-infected A549 cells at 24 hpi using NP and FLAG antisera revealed NP and GP-1 distribution was distinct; while NP was distributed widely in perinuclear regions with occasional dense puncta, GP-1 was mostly localized on one side of the nucleus, reminiscent of Golgi distribution (Fig. 4A). To confirm Golgi localization of GP-1, we performed IF using antisera specific for both FLAG and the *cis*-Golgi marker GM130, for which many peak intensities spatially corresponded, quantified by line scan analysis across a distance of approximately 20 µm (Fig. 4B). This confirmed previous localization studies that used transient LCMV GPC expression (39), although here, we report for the first time the cellular location of GP-1 expressed from an infectious LCMV.

### rLCMV infection results in redistribution of COPI complex components

Using rLCMV-GP1-FLAG, we next examined the spatial proximity of NP and GP-1 alongside components of the COPI complex.

**Fig 5:**
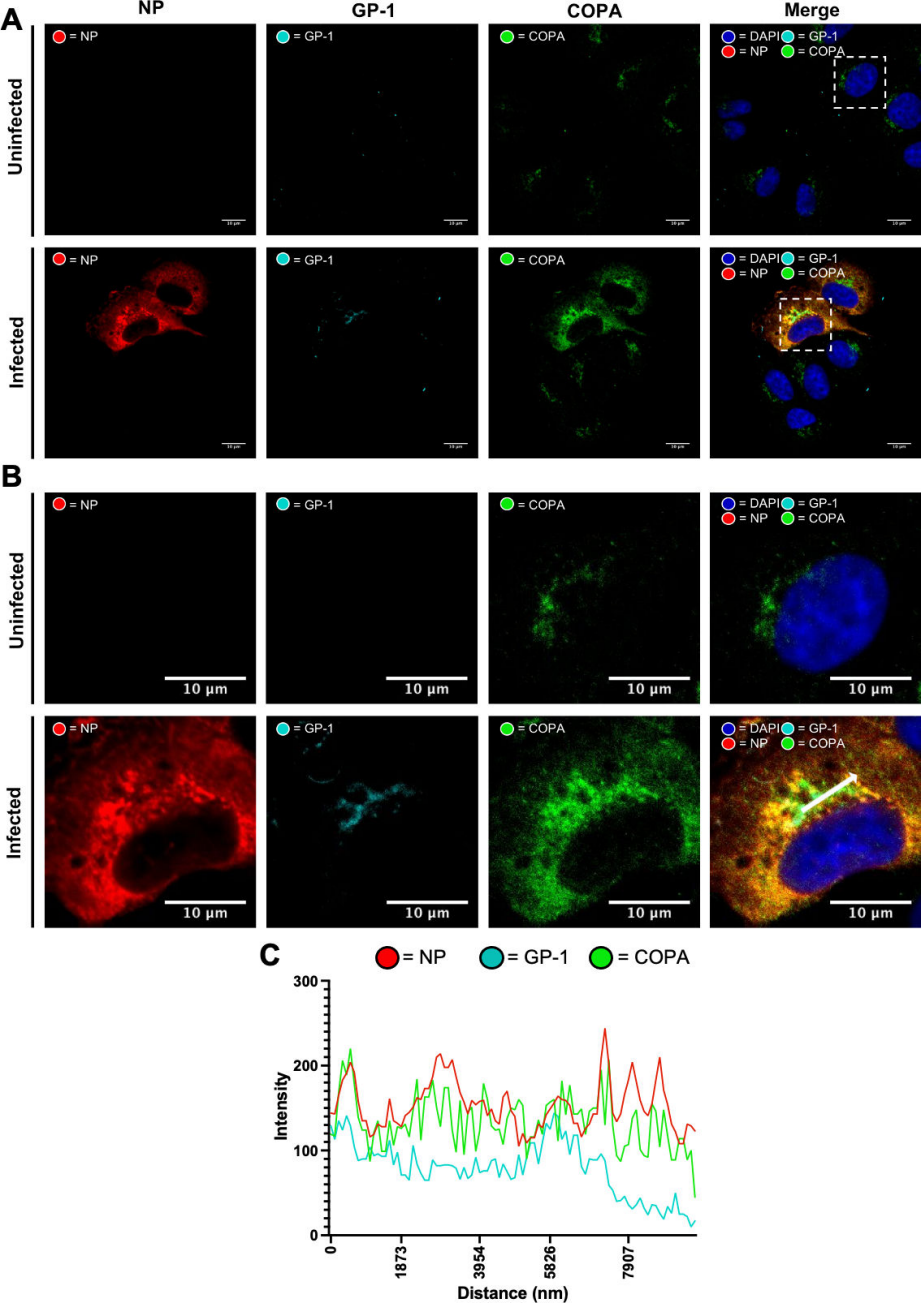
LCMV NP and the COPA component of COPI complexes co-localize in LCMV-infected A549 cells. (**A**) Confocal microscopy of uninfected and rLCMV-GP1-FLAG-infected cells at 15 hpi, MOI of 0.1. A549 cells were stained with antisera specific for COPA (green), GP-1 (cyan), and NP (red) alongside DAPI. (**B**) A zoomed image for both uninfected and infected (white boxes) for all channels is shown, alongside merged channels. (**C**) Line scan for the region highlighted in the zoomed image in panel (B) (scan line represented by a white arrow), showing intensities of channels corresponding to NP, GP-1, and COPA across a ~10 µm distance.

As expected, native COPA staining within uninfected cells presented as discrete puncta with characteristic ER/Golgi distribution (Fig. 5A and B). In contrast, within rLCMV-GP1-FLAG-infected cells, a portion of COPA staining exhibited this same native ER/Golgi localization, but in these infected cells COPA staining was also detected outside perinuclear regions, extending throughout the cytoplasm. This redistributed COPA staining closely corresponded with that of NP, which is represented in the merged image and associated line scan (Fig. 5C). To examine whether COPA and NP interacted directly, we immunoprecipitated LCMV NP from infected cells and performed mass spectrometry analysis of the eluate, however COPA was not identified, suggesting any interaction between NP and COPA was indirect (data not shown).

Co-staining with FLAG antisera revealed GP-1 occupied a restricted area within that occupied by COPA, with many of the peak intensities of GP-1 and COPA being closely aligned, as shown by line scan analysis (Fig. 5C). This GP-1 and COPA colocalization was consistently imaged across all infected cells analyzed (Fig. S1A). As with our previous observations, described above (Fig. 4A and B), NP and GP-1 occupied distinct regions, and the positions of their peak intensities or general abundance did not precisely correspond.

Taken together, these findings suggest a close association between COPA and NP, as well as between COPA and GP-1, consistent with a role for COPI in LCMV NP and GPC functions.

### rLCMV infection results in redistribution of AP-4 complex components

**Fig 6:**
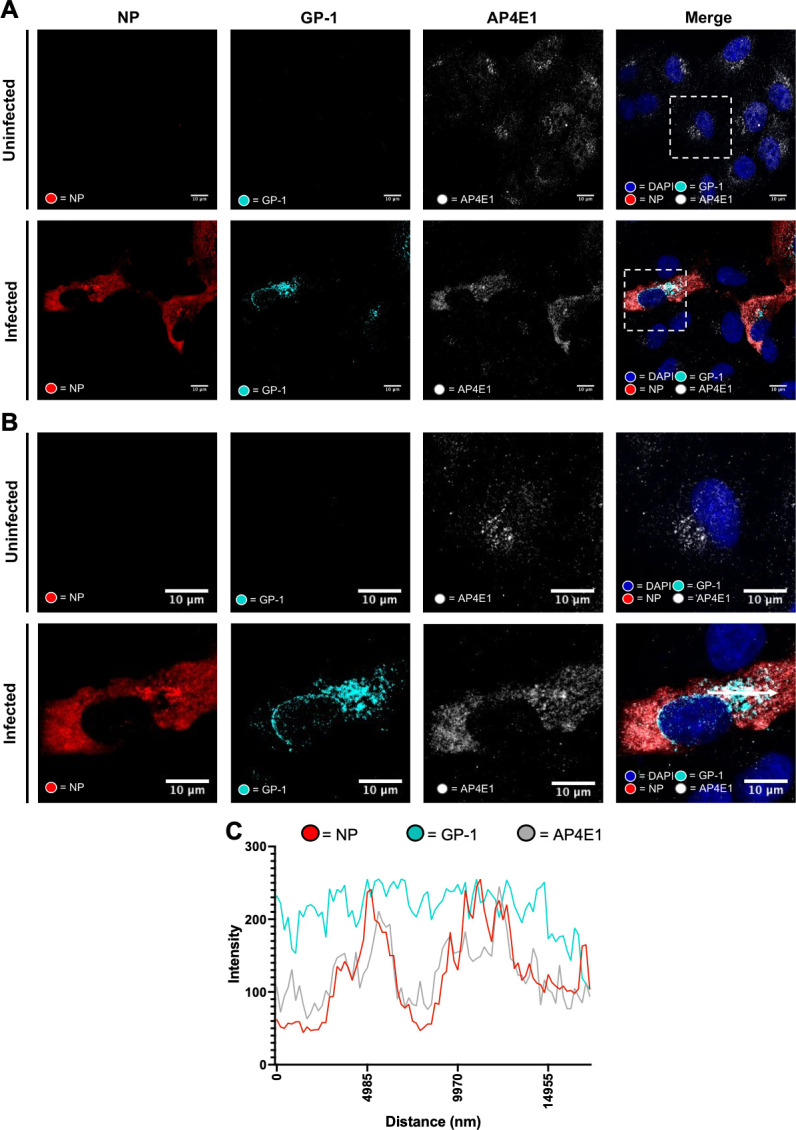
LCMV NP and the AP4E1 component of AP-4 complexes co-localize in rLCMV-GP1-FLAG-infected A549 cells. (**A**) Confocal microscopy of uninfected and rLCMV-GP1-FLAG infected cells at 15 hpi, MOI of 0.1. A549 cells were stained with AP4E1 (white), GP-1 (cyan), and NP (red) and DAPI. (**B**) A zoomed image for regions of both uninfected and infected cells (white boxes) for all channels is shown, alongside merged channels. (**C**) Line scan analysis for the region highlighted in the merged zoomed image shown in panel (B) (scan line represented by a white arrow) showing intensities of channels corresponding to NP, GP-1, and AP4E1 across a ~17 µm distance.

As with components of the COPI complex described above, rLCMV-GP1-FLAG allowed examination of the spatial proximity of NP and GP-1 alongside components of the AP-4 complex. Due to the unavailability of AP4M1 antisera suitable for IF analysis, we instead used antisera for AP-4 subunit AP4E1 (Fig. 6A and B). When used to stain uninfected cells this antisera revealed AP4E1 was located within the *trans*-Golgi network, consistent with previous reports (37, 38). In contrast, within rLCMV-GP1-FLAG-infected cells, the majority of AP4E1 was located diffusely throughout the cytoplasm, suggestive of redistribution.

Staining of rLCMV-GP1-FLAG-infected cells with NP antisera revealed close colocalization with AP4E1, suggesting NP was responsible for the observed AP4E1 redistribution (Fig. 6C). In contrast, while GP-1 staining occupied a portion of that occupied by AP4E1/NP, its distribution was less extensive and more reminiscent of Golgi, as described above. To gain a better overall representation of GP-1, NP and AP4E1 distribution, colocalization of all these components was imaged in multiple different rLCMV-GP1-FLAG-infected cells (Fig. S1B), which revealed a consistent outcome.

Taken together, these findings suggest AP4E1 is redistributed during LCMV infection adopting a localization that closely matches that of NP, consistent with a role for AP-4 complexes during the viral multiplication cycle.

#### BFA treatment of LCMV-infected cells during single-step growth does not significantly affect gene expression or genome RNA replication

To further investigate the role of COPA and AP-4 complexes in the LCMV replication cycle, we used brefeldin-A (BFA), which inhibits ARF-1 activation, blocking the formation of COPI and AP-4 complexes (40, 41). We determined that BFA was non-toxic for human-origin A549 cells in concentrations up to 5,000 ng/mL (Fig. S2A), at which NP and eGFP expression were impacted during multi-step growth measured at 24 hpi (Fig. S2B through D). As a positive control, we also showed BFA efficacy against influenza A virus (IAV) gene expression, measured by expression of IAV NP and eGFP expression from a GFP-tagged IAV, previously shown to be diminished by BFA treatment (33, 42).

We confirmed BFA resulted in the expected cellular changes by staining BFA-treated cells with antisera specific for COPA and AP4E1 components. Under normal cellular conditions, both COPA and AP4E1 localized in discrete perinuclear regions, consistent with Golgi/TGN localization (Fig. S3). However, upon the addition of 5,000 ng/mL BFA for 1 h, both COPA and AP4E1 staining became diffusely distributed throughout the cytoplasm.

**Fig 7:**
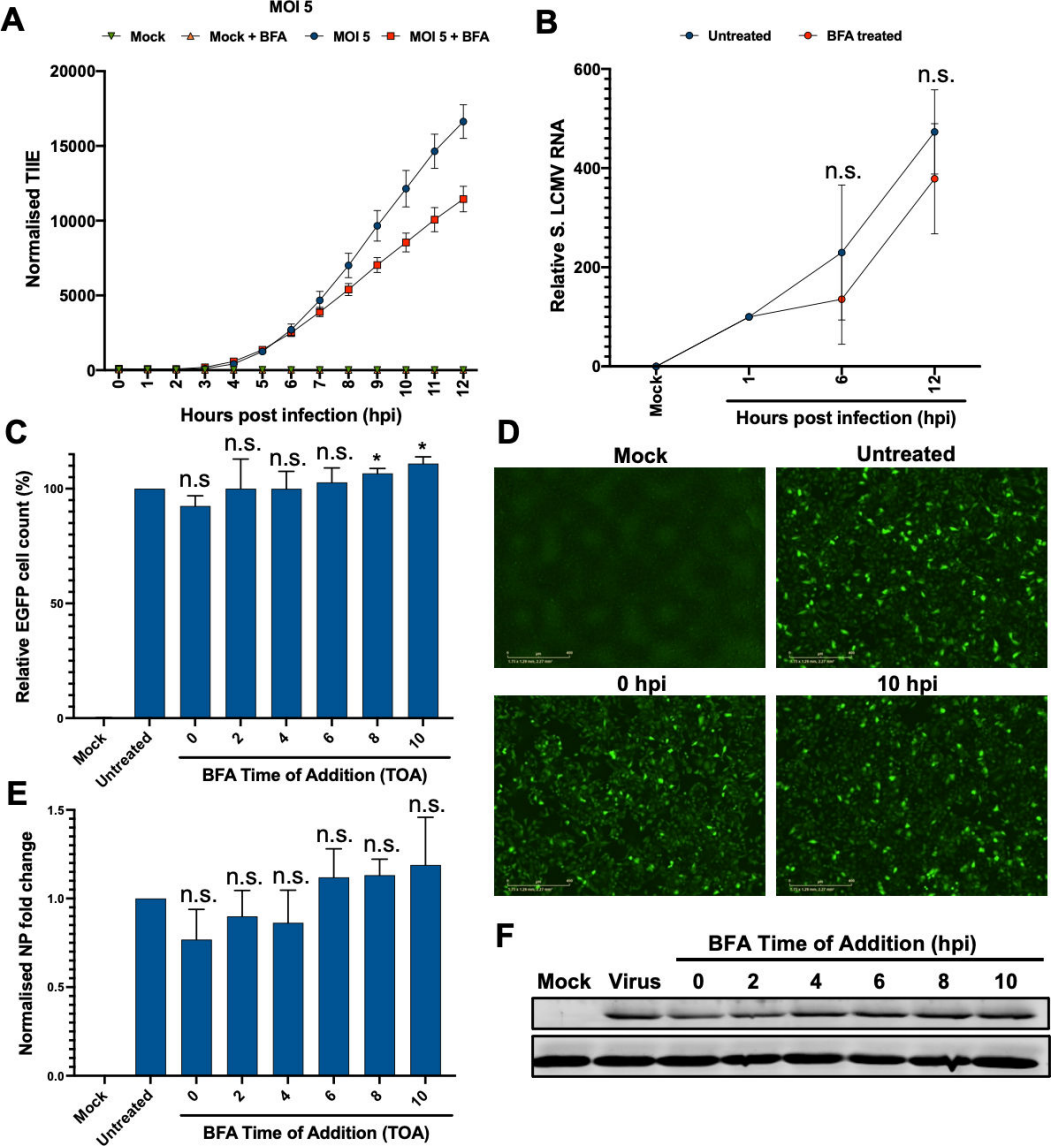
Temporal analysis of LCMV gene expression and vRNA synthesis suggests BFA treatment has little impact of LCMV gene expression. (**A**) A549 cells were pre-treated with BFA (5,000 ng/mL) and then infected with rLCMV-eGFP at an MOI of 5. At hourly intervals, TIIE was quantified, normalized for confluency over three experimental repeats, with error bars showing deviation from the mean. (**B**) A549 cells were pre-treated with BFA (5,000 ng/mL) and then infected with rLCMV-eGFP at an MOI of 5. At the indicated time points, the cells were harvested for RNA extraction and RT-qPCR used to quantify S segment vRNA at each time point, normalized to the respective 1 hpi control. Four experimental repeats were performed, with error bars showing deviation from the mean. (**C**) For time of addition assays, A549 cells were infected with rLCMV-eGFP at an MOI of 5, with BFA added at bi-hourly intervals and remaining present for the duration of a single round of infection. eGFP cell count was quantified, normalized to the untreated control, with three experimental repeats represented, with error bars showing deviation from the mean. (**D**) Live-cell fluorescence images of with mock, untreated, BFA added at 0 hpi and 10 hpi shown as examples. (**E**) Alongside, cell lysates were probed using antisera for LCMV NP and GAPDH as a loading control with band intensities quantified using densitometry and plotted showing relative fold-change of NP expression over three experimental repeats with error bars showing deviation from the mean, with a representative western blot shown in (**F**).

To confirm the involvement of ARF-1 and GBF-1 in LCMV multiplication, we next examined the effect of BFA on LCMV gene expression over a single cycle of LCMV infection, which we have previously determined to take 10–12 h, when infectious virions are first released (32). A549 cells were pre-treated for 45 min with BFA at 5,000 ng/mL prior to infection with rLCMV-eGFP at an MOI of 5, chosen to facilitate detection of viral activities at early time points. To measure rLCMV-eGFP gene expression, TIIE was measured hourly, with BFA addition initially showing no significant impact on eGFP expression at times up to 7 hpi, and causing a small but significant drop in eGFP expression between 8 and 12 hpi of around 0.25-fold (Fig. 7A).

Next, to determine whether BFA treatment impaired LCMV RNA synthesis, we quantified S segment vRNA abundance during a single round of infection, as above, achieved by limiting the time frame of analysis up to 12 hpi. rLCMV-eGFP-infected cells were harvested for RNA extraction at 1, 6, and 12 hpi, with S segment vRNA abundance determined by qRT-PCR analysis, with the 1 hpi time point used to normalize variation in input vRNA quantity. This analysis revealed no significant difference in S segment vRNA abundance for any time point analyzed between BFA treated and untreated samples [S vRNA; 6 hpi (*P* = 0.104729); 12 hpi (*P* = 0.061214)] (Fig. 7B). Thus, we conclude that BFA has no detectable effect on vRNA production within a single round of infection.

#### Time-of-addition experiments suggest BFA treatment has no significant impact on LCMV NP production

To corroborate our finding that BFA treatment had little or no impact on either LCMV gene expression or RNA synthesis, we performed time-of-addition (TOA) experiments, with BFA added to cultures at various times post-infection. The cells were infected with LCMV-eGFP with an MOI of 5, then treated with BFA at time points of 0, 2, 4, 6, and 10 hpi, with sampling restricted to a 12-hpi window, chosen to span a single LCMV multiplication cycle (32), as above (Fig. 7A and B). Impact of BFA treatment was first assessed by determining the eGFP cell count within infected cultures, measured by live-cell fluorescence imaging and normalized for confluency over three independent experimental repeats (Fig. 7C and D), with BFA treatment causing no significant decrease in eGFP expression.

As an orthogonal approach, we also assessed NP expression from TOA cultures (MOI of 5), with all lysates harvested at 12 hpi regardless of BFA treatment times and analyzed by Western blotting using NP antisera. Quantification of three independent experimental repeats (Fig. 7E and F) revealed that LCMV NP expression was not significantly diminished by BFA treatment at any of the time points, even when BFA was present from the entire experimental period, from 0 hpi (Fig. 7E; compare untreated and 0 hpi data points). Thus, the addition of BFA at temporally distinct phases of a single LCMV infection cycle had no significant impact on gene expression.

### BFA treatment inhibits infectious virus assembly and release

**Fig 8:**
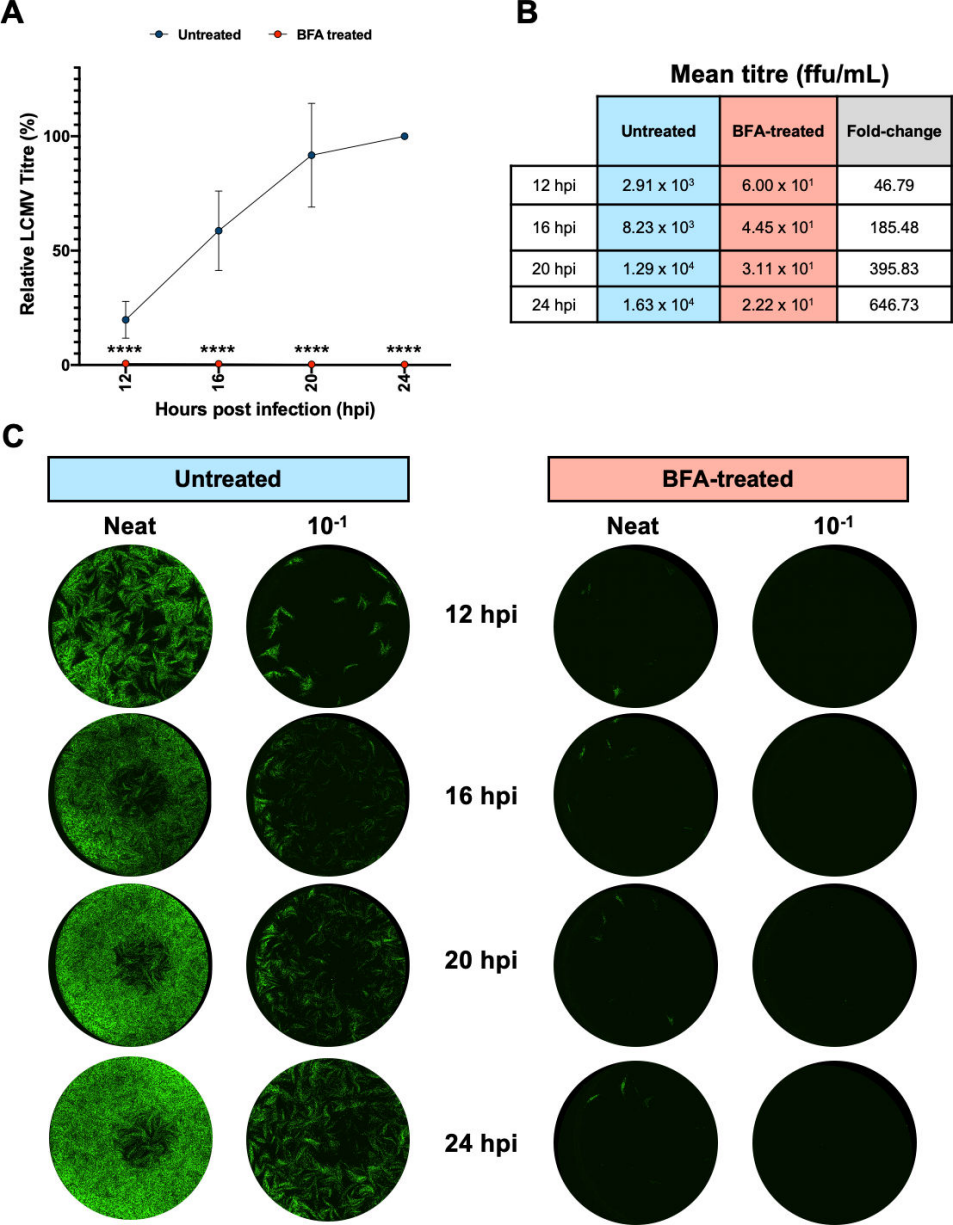
BFA treatment has a major impact on LCMV growth. (**A**) A549 cells were either untreated or pre-treated with BFA (5,000 ng/mL) and then infected with rLCMV-eGFP at an MOI of 5. At the indicated times post-infection, viral supernatants were collected and subjected to titration via FFA, with titers from three experimental repeats plotted alongside the indicated time point, normalized to the 24 hpi untreated control, with error bars showing deviation from the mean. (**B**) Tabulated mean viral titers (ffu/mL) for three independent experimental repeats for supernatants harvested at 12, 16, 20, and 24 hpi, for both untreated (blue) and BFA-treated (red) conditions, with the mean fold-change ratio of untreated/BFA-treated, also shown. (**C**) Representative images of FFA wells, for both untreated and BFA-treated cultures, at dilutions of neat, and 10^−1^.

The results presented above showed BFA treatment of rLCMV-eGFP-infected cells resulted in no significant change in LCMV NP expression (Fig. 7E and F). Next, the effect of BFA treatment on infectious virus release were assessed. As for the previous experiments where gene expression was examined, we wanted to assess virus release during a single round of infection only. To achieve this, we performed a classical virus growth experiment with A549 cultures infected with LCMV-eGFP at an MOI of 5, with or without BFA pre-treatment, with supernatants harvested at 12 hpi when infectious virus is first released (32). Supernatants were also harvested at additional 16, 20, and 24 hpi time points for completeness, and viral titers calculated by FFA represented relative to the untreated 24 hpi sample (Fig. 8A), with the actual titers tabulated alongside (Fig. 8B). In BFA-treated cultures (Fig. 8A; red data points), the titers at all time points were consistently less than 1% of the untreated control (Fig. 8A; blue data points) [BFA viral titers; 12 hpi 0.62% (*P* = 0.000006872); 16 hpi 0.49% (*P* = 0.000004219); 20 hpi 0.26% (*P* = 0.000000364); 24 hpi 0.21% (*P* = 0.000000316)] thus showing BFA treatment exerts major influence on virion production, with mean titers reduced by over 46-fold at the 12 hpi time point increasing to over 646-fold at 24 hpi (Fig. 8B), with representative FFAs shown in Fig. 8C.

Taken together, these findings suggested that LCMV gene expression and vRNA production were not significantly affected by BFA treatment. In contrast, BFA treatment exerted major influence on the production of infectious virus, suggesting an important role for COPI and AP-4 complexes in this process.

## DISCUSSION

Here, we used rLCMV-eGFP for identification of host cell trafficking factors required for LCMV multiplication, with measurement of virus-specific eGFP expression allowing high-throughput screening of siRNA-mediated gene knockdown of host cellular genes on various stages of the LCMV multiplication cycle.

The siRNA screen comprised 261 siRNAs, specific for 87 target genes, and revealed an important role for multiple cellular factors, of which multiple components of AP-4 and COPI coatomer complexes were highly ranked, judged by the impact of expression knockdown on virus-specific eGFP expression (Fig. 1). Corroborating the importance of COPI and AP-4 components, ARF-1, and GBF-1, required by both AP-4 and COPI complexes for anchorage to Golgi membranes (43), were also high-ranking in terms of LCMV gene expression knockdown. AP4M1 is one of four components of the AP-4 complex that perform native cellular roles in trafficking of cargo proteins from the TGN to the plasma membrane (38), whereas COPA is one of seven components of the COPI complex, with well-characterized involvement in Golgi-ER retrograde transport and intra-Golgi trafficking, but also the expression of cell surface receptors and maturation of early endosome to autophagic vacuoles (44). We note that antisera suitable for optimal detection of all these subunits are not currently available, which had some influence on our experimental designs. For example, due to the difficulty in sourcing an AP4M1 antibody that was suitable for IF analysis, we instead used antisera against component AP4E1 to identify AP-4 complex location.

To gain a better understanding of AP-4 and COPI involvement in the LCMV multiplication cycle, we generated a recombinant LCMV mutant rLCMV-GP1-FLAG containing a FLAG-tagged epitope within GP-1, which alongside our existing NP antisera allowed the spatial cellular distribution of these viral components to be examined alongside AP-4 and COPI complexes within virus-infected cells. First, using this virus, we showed that LCMV GP-1 was predominantly located within the Golgi compartment. This was distinct from NP staining, which was dispersed more widely throughout the cytoplasm, while also sharing some Golgi localization.

Next, we examined the cellular localization of NP and GP-1 alongside AP-4 complex components. This showed virus infection led to redistribution of AP4E1, and by inference AP-4 complexes, away from its characteristic *trans*-Golgi location, to occupy regions of the cell where GP-1 and in particular NP was most abundant, suggestive of a close interaction between these components. Selection of AP-4 cargo is known to be mediated through the binding of a YXXØ or dileucine-based motifs [(D/E)XXXL(L/I) and (LL/LI)] within the cytoplasmic tail of cargo proteins to the AP4M1 subunit (38, 45). LCMV NP has three YXXØ and 11 LCMV dileucine-based motifs; GPC has six YXXØ and 10 dileucine-based motifs, although none lie within the predicted cytoplasmic tails, despite these tail domains being crucial in GP-1/GP-2 trafficking to the plasma membrane (46). One possibility is that NP-mediated redistribution of AP-4 could be mediated by components of the GPC, for instance, during transport of partially assembled RNPs in association with GPC components to a plasma membrane assembly site.

Currently, there are many reports of viral subversion of members of the adapter protein complexes (AP1-5), although there are few reports of viral roles specifically for the AP-4 complex (39, 47). In the case of hepatitis C virus (HCV), mutational studies showed that the HCV NS2 protein binds to the AP4M1 subunit via its dileucine motifs. When these motifs were mutated, HCV RNA replication was not impaired, yet extracellular viral titers were reduced (45). This study also implicated AP-4 in promoting direct cell-to-cell spread (45). Another example of viral AP-4 subversion is for Epstein-Barr virus (EBV) of the *Herpesviridae* family, which encodes a transmembrane glycoprotein BMRF-2 that has previously been shown to interact with the µ subunit of AP-4, permitting BMRF-2 trafficking from the TGN to the basolateral plasma membrane and thus EBV egress (48).

This study also focussed on the COPI coatomer complex, with knockdown of two of its substituent components, COPA and COPB1, resulting in an approximately 50% reduction in LCMV-mediated eGFP expression within the siRNA screen protocol. Although components of COPI complexes have previously been described as important for the LCMV replication (49), this is the first report of COPI component involvement in a neuronal cell line that is relevant to the LCMV replication cycle. As described above for AP-4, using rLCMV-GP1-FLAG allowed the examination of GP-1 and NP localization in relation to COPI components. This analysis revealed a high degree of colocalization between COPA and NP in both dense puncta and also a more dispersed cytoplasmic distribution, as well as close colocalization between GP-1 and COPA, although within a more restricted region, characteristic of the Golgi. Taken together, these findings are entirely consistent with COPI components playing important roles in virion assembly and egress, corroborating the results of the siRNA knockdown analysis.

Also supportive of a role for COPI in LCMV multiplication was the finding that BFA pre-treatment of infected cells resulted in no significant reduction in LCMV NP expression, within a single infectious cycle (Fig. 7E) but almost 50-fold reduction in infectious virion production over the same period (Fig. 8A and B), indicating a role for COPI and AP-4 complexes in the release of infectious virus. It is well-established that LCMV GPC is cleaved to GP-1/GP-2/SSP trimers by host cell protease SKI-1/S1P within the Golgi (26) and so one possibility is that canonical COPI complexes are utilized for intra-Golgi and retrograde Golgi-ER trafficking (50) that may be required for efficient GPC proteolytic processing.

The use of siRNA mediated depletion or BFA treatment has provided many examples where COPI components are required for efficient virus multiplication (44), with many examples from within the *Bunyavirales* order. We have previously reported a reliance on COPI complexes and ARF-1 involvement for the bunyavirus Hazara virus (HAZV), which is a species within the *Nairoviridae* family (33), a group that share many genetic and functional characteristics with the arenaviruses (51). We showed that HAZV requires COPI complexes in both ARF-I dependent and ARF-I independent processes, at early and late stages of the multiplication cycle (33), respectively. Particularly prominent was an influence of BFA on infectious virus production, similar to our current findings for LCMV. In addition to this, and likely related to COPI complex involvement, Uukuneimi virus, a member of the *Phenuiviridae* family within the *Bunyavirales* order, has been shown to require GBF-1 activation, and thus ARF-1 activation, for viral replication and particle assembly (52). It is therefore possible that COPI complex involvement is a common requirement for all bunyaviruses. Whether this relates to a critical reliance on entirely functioning Golgi compartment for the formation of the bunyaviral factory, or a more general requirement for the formation of other lipid-based compartments that depend on COPI complex activity is an interesting topic for future research (53, 54).

Collectively, our findings improve the understanding of arenaviruses host-pathogen interactions and reveal the involvement of important cellular trafficking pathways required during infection. Moreover, this study may lead to the discovery of novel therapeutic targets for arenaviruses to prevent serious human disease.

## MATERIALS AND METHODS

### Plasmid design

Plasmids for rLCMV-WT and rLCMV-eGFP viral rescue were previously generated and described (32). rLCMV-GP1-FLAG plasmid was generated by first performing sequence alignment of GP-1 alongside closely related OW arenaviruses to select a location for FLAG tag insertion. The predicted structure of GP-1 for rLCMV-GP1-FLAG was investigated using SWISS-MODEL and mapped alongside the solved LCMV GP-1 structure, which showed the FLAG insertion site to be within an unsolved flexible loop of GP-1, likely accessible for antibody recognition. rLCMV-GP1-FLAG was generated by insertion PCR utilizing Q5 site-directed mutagenesis kit (New England BioLabs) and FLAG primers, according to the manufacturer’s instructions. Positive colonies were verified via colony PCR and DNA sequencing (Genewiz).

### Recovery of rLCMV-WT, rLCMV-eGFP, and rLCMV-GP1-FLAG

Recovery of infectious variants of LCMV has previously been described (32), based on the work of the de la Torre group (55). In brief, six-well plates were seeded with 2 × 10^5^ BSR-T7/well 1 day prior to transfection in 2 mL of Dulbecco modified Eagle medium (DMEM) supplemented with 2.5% fetal bovine serum (FBS), 100 U/mL penicillin, and 100 µg/mL streptomycin (2.5% DMEM). After 24 h, the cells were transfected with 1.6 µg pUC57-S-WT, 1.6 µg pUC57-NP (+), 2.8 µg pUC57-L, 2.0 µg pUC57-L (+), and 0.6 µg pCAG-T7pol, combined with 2.5  µL of Mirus TransIT-LT1 transfection reagent (Mirus Bio) per μg of DNA, in 200 µL of Opti-MEM. For rLCMV-eGFP recovery, the WT plasmid was replaced with pUC57-S-eGFP. A control sample, in which pUC57-L and pUC57-L (+) were omitted, was included for each virus recovery experiment. At 24 h post-transfection (hpt), media containing the transfection mix was removed and replaced with fresh 2.5% DMEM. Reinfection of fresh monolayers was carried out in six-well plates seeded with 2  ×  10^5^ BHK cells/well 1  day prior to infection in 2 mL of DMEM supplemented with 2.5% FBS, 100 U/mL penicillin, and 100 µg/mL streptomycin. Fresh BHK cells were washed with phosphate-buffered saline (PBS) two times prior to infection. Supernatants from transfected BSR-T7 cells were collected at 120 hpt, centrifuged at 4,000 × *g* for 15 min and 1 mL used to infect fresh BHK cells in a six-well plate in DMEM with 2.5% FBS. Cell supernatant from infected BHK cells was collected at 72 h post-infection (hpi), centrifuged at 4,000 *× g* for 15 min and viral stocks were stored as aliquots at −80°C. This protocol was followed for the successful recovery of the newly developed mutant rLCMV-GP1-FLAG, with the S plasmid of LCMV being replaced with the recently generated pUC57-RQ-S-FLAG. The viral supernatant gained from successful infection following transfection was centrifuged at 4,000 *× g*, aliquoted (80 µL) and frozen for subsequent viral titration and bulking.

### Virus infections

For the generation of a bulk stock of rLCMV-GP1-FLAG, a T175 flask was seeded with 5 × 10^6^ BHK-21 cells 1 day prior to infection at an MOI of 0.001. At 3 days post-infection, viral supernatant was harvested and centrifuged at 4,000 *× g* to remove cell debris, aliquoted (80 µL) and frozen for subsequent viral titration. For viral infections, cell monolayers were infected with LCMV at the specified MOI in either serum-free (SFM), 2.5% or 10% FBS DMEM, depending on cellular requirements, at 37°C. After 1 h, the inoculum was removed and SFM, fresh 2.5% or 10% DMEM was then applied for the duration of the infection. For synchronized infections, LCMV was incubated on ice for 1 h to facilitate adsorption. Subsequently, the inoculum was removed, monolayers were washed three times with PBS and SFM, fresh 2.5% or 10% DMEM was then applied for the duration of the infection.

### Viral titration

Virus titers were determined by focus-forming assays. Viral stocks requiring titration were serially diluted in SFM to infect fresh monolayers of BHK cells seeded at 1 × 10^5^ in a 24-well plate and incubated at 37°C for 1 h. After infection, medium-containing virus was removed and 2 mL of overlay containing 10% FBS DMEM and 1.6% methylcellulose at a 1:1 ratio was reapplied to cells, then incubated for a further 3 days at 37°C. For rLCMV-eGFP titration, whole-well images were taken using an Incucyte S3 live-cell imaging system (Sartorius) and rLCMV-eGFP foci were then counted and virus titers were determined as focus-forming units/mL (FFU/mL). For rLCMV-WT titration, the cells were fixed using 4% (vol/vol) paraformaldehyde (PFA) for 15 min and washed three times with PBS. The cells were then incubated with permeabilization buffer [0.1% (vol/vol) Triton X-100, 2% (wt/vol) FBS in 1× PBS] for a further 15 min and washed three times with PBS. After permeabilization, the cells were incubated with 1 mL blocking buffer [2% (wt/vol) FBS in PBS] for 1 h, then incubated for 1 h with 150 µL/well LCMV NP primary antibody (in-house, 1:1,000 in blocking buffer), and washed three times with PBS. Following this, the cells were incubated for 1 h with 594 Alexa Fluor secondary antibody (Life Technologies; 1:500 in blocking buffer) and washed four times with PBS. The Incucyte S3 live-cell imaging system (Sartorius) was then used to image whole wells of the plate to detect red rLCMV-WT foci, which were counted, and virus titers determined. For rLCMV-FLAG titration, the cells were also stained with 150 µL/well FLAG (Sigma, 1:500 in blocking buffer) primary antibody and 488 (FLAG) Alexa Fluor secondary antibody (Life Technologies; 1:500 in blocking buffer), then washed and imaged as described above.

### Reverse transfection of siRNA library

Trypsinized SH-SY5Y cells in 10% FBS DMEM were counted using a haemocytometer and used to make a cell suspension containing 1 × 10^5^ cells/mL. A master mix was made resulting in 0.3 µL of Lipofectamine RNAiMAX reagent (Invitrogen) and 16.7 µL of Opti-MEM per well and 17 µL of this master mix were pipetted into each well of a 96-well plate. All siRNAs were taken from the “silencer” human membrane trafficking siRNA library (thermo). A 3 µL volume of working stock siRNA (1 µM) was pipetted into the transfection master mix and mixed, resulting in a final concentration of 3 pmol of siRNA per well. Transfection master mix and siRNA were incubated for 20 min, following which 1 × 10^5^ cells in 10% DMEM (100 µL total volume) were then applied per well. The cells were incubated with the transfection and siRNA mix for 24 h at 37°C, following which 60 µL of the medium was removed and replaced with 200 µL of fresh 10% FBS DMEM to dilute out any potential toxic effects of the siRNAs or transfection reagent. At 6 h post-dilution, the medium was removed, and the cells were washed in PBS prior to infection with rLCMV-eGFP at an MOI of 0.2 in 100 µL of 10% FBS DMEM for 1 h. Following infection, virus was removed, and the cells were washed two times with PBS to remove any unbound virus, then supplemented with 200 µL 10% FBS DMEM. At 24 hpi, eGFP fluorescence intensity was determined using the Incucyte S3 live-cell imaging software as a measure of virus multiplication. The total integrated intensity of eGFP (TIIE; green count units [GCU] × μm^2^/image) was first normalized to confluency per well and then analyzed as a percentage of the total green integrated intensity in positive control wells containing virus and lipofectamine but omitting siRNA. Normalized values were averaged between four experimental repeats.

### Immunofluorescence (confocal microscopy)

Trypsinized A549 cells were seeded onto a 19-mm round glass coverslip (VWR) in a 12-well plate at 1 × 10^5^ cells/well, followed by incubation at 37°C. After 14 h, BFA was added to cells (5,000 ng/mL) for 1 h prior to fixation. For IF (omitting AP4E1, see below), the cells were washed two times in PBS prior to fixation in 4% (vol/vol) paraformaldehyde in PBS for 15 min at room temperature. After fixation, the cells were washed three times in PBS and then incubated in permeabilization buffer (0.1% [vol/vol] Triton X-100, 1% [wt/vol] bovine serum albumin [BSA] in PBS) for 15 min at room temperature. Following permeabilization, the monolayers were washed three times with PBS and incubated with blocking buffer [1% (wt/vol) BSA in PBS] for 1 h. Subsequently, primary antibody was diluted in BSA blocking buffer as follows: NP [1:500, in house (sheep)], FLAG [Sigma; 1:250 (mouse)], and COPA primary antibody [GeneTex; 1:100 (rabbit)] and incubated for 1 h at room temperature. The cells were then washed three times with PBS and incubated with corresponding Alexa Fluor 488, 594 and 647 secondary antibodies (Life Technologies; 1:500 in BSA blocking buffer) for 1 h at room temperature in a light-protected vessel. Cell monolayers were then washed three times with PBS and mounted onto glass coverslips with the addition of Prolong Gold Antifade reagent with DAPI (Thermo Fisher Scientific), cured, sealed, and stored at 4°C. Images were then taken on an LSM 880 confocal microscope (Zeiss) and processed using Zen (Blue Edition) software and Fiji (ImageJ). Line scan analysis was performed utilizing Zen (Blue Edition).

For AP4E1 IF, the cells were washed two times with PBS and fixed with 100% methanol for 5 min on ice. After fixation, the cells were washed three times in PBS and then incubated with blocking buffer [0.1% (wt/vol) Saponin, 1% (wt/vol) BSA in PBS] for 30 min. Subsequently, primary antibodies diluted in saponin/BSA blocking buffer containing NP [1:500, in house (sheep)], FLAG [Sigma; 1:250 (rabbit)], and AP4E1 primary antibody [BD Biosciences; 1:75 (mouse)] was incubated for 1 h at room temperature. The cells were then washed three times with PBS and incubated with corresponding Alexa Fluor 488, 594, and 647 secondary antibodies (Life Technologies; 1:500 in saponin/BSA blocking buffer) for 1 h at room temperature in a light-protected vessel. Cell monolayers were then washed three times with PBS and mounted onto glass coverslips with the addition of Prolong Gold Antifade reagent with DAPI (Thermo Fisher Scientific), cured, sealed, and stored at 4°C. Images were then taken on an LSM 880 confocal microscope and processed as described above.

### Inhibition of retrograde transport

Trypsinised A549 cells were seeded into 24-well plates at 1 × 10^5^ cells/well and incubated at 37°C. After 16–24 h, the cells were pre-treated with BFA at the indicated concentration for 45 min in SFM. Following this, rLCMV, rLCMV-eGFP, A/WSN/33 H1N1 (denoted as rIAV herein), and rIAV-eGFP was added directly to each well at an MOI of 0.1. For WT viruses, 24 hpi cells were lysed for analysis via western blotting. Densitometry was performed using Fiji software and NP expression normalized to confluency per well, then analyzed as a percentage of virus only DMSO control. Normalized values were averaged between three biological repeats for WT viruses (*n* = 3). For eGFP viruses, at 24 hpi, the Incucyte S3 live-cell imaging system (Sartorius) was utilized to measure eGFP fluorescence. The total integrated intensity of eGFP (TIIE; green count units [GCU] × μm^2^/image) was first normalized to confluency per well and then analyzed as a percentage of the total green integrated intensity in virus only DMSO control. Normalized values were averaged between three biological repeats for eGFP mutant virus (*n* = 3). For titration following BFA treatment, viral supernatant was collected for mock, virus, 50, 500, and 5,000 ng/mL. Focus-forming assays were performed for each BFA condition as previously described for LCMV-eGFP (*n* = 3).

### Temporal kinetic analysis of inhibition of retrograde transport

For gene expression analysis, trypsinized A549 cells were seeded into 12-well plates at 1 × 10^5^ cells/well and incubated at 37°C. After 16–24 h, the cells were pre-treated with BFA at 5,000 ng/mL for 45 min. Following this, rLCMV-eGFP was added directly to each well at an MOI of 1 and 5. Incucyte S3 live-cell imaging system (Sartorius) was utilized to measure eGFP fluorescence at hourly intervals. The total integrated intensity of eGFP (TIIE; green count units [GCU] × μm^2^/image) was first normalized to confluency per well and then analyzed as a percentage of the total green integrated intensity for untreated 24 hpi control. Normalized values were averaged between three biological repeats (*n* = 3).

For qPCR analysis, A549 cells were seeded into a 12-well plate at 1.5 × 10^5^ cells/well. Following 16–24 h, the cells were pre-treated with BFA at 5,000 ng/mL for 45 min. Subsequently, drug media were collected, and the cells were infected at an MOI of 5 with rLCMV-WT in minimum volume 10% FBS DMEM media (250 µL) for 1 h. Following this, infection media were removed, and monolayers were washed four times with 1× PBS, and drug media were replaced. At each indicated time point, cells lysates were washed with 1× PBS, trypsinized,and pelleted (500 × *g*) for 5 min. RNA extraction was performed following the manufacturer’s instructions for Monarch Total RNA Miniprep Kit (New England BioLabs). Two-step qPCR was carried out firstly by converting RNA into cDNA following the manufacturer’s instruction for LunaScript RT SuperMix Kit (New England BioLabs). Subsequently, qPCR was performed using the cDNA following the manufacturer’s instructions for Luna Universal qPCR Master Mix. Analysis of S segment vRNA expression via qPCR for mock and virus (MOI = 5) in untreated and BFA-treated conditions was performed, alongside GAPDH as the housekeeping control. The primer sequences used were as follows: S vRNA (5′-CAG CCA ACA ACT CCC ACC AT-3′ and 5′-GAA GGC AGA GGT CAG ATT GCA-3′) and GAPDH (5′-TCA CCA CCA TGG AGA AGG CT-3′ and 5′-GCC ATC CAC AGT CTT CTG GG-3′). vRNA levels, for each time point, in untreated and BFA treated were normalized for their respective input (1 hpi). Normalized values were averaged between four experiment repeats.

For viral titration, A549 cells were seeded into a 12-well plate at 1 × 10^5^ cells/well. Following 16–24 h, the cells were pre-treated with BFA at 5,000 ng/mL for 45 min. Subsequently, drug media were collected, and the cells were infected at an MOI of 5 with rLCMV-eGFP in minimum volume of 10% FBS DMEM media (250 µL) for 1 h. Following this, infection media were removed, and monolayers were washed four times with 1× PBS, and drug media were replaced. At each indicated time point, viral supernatants were collected and titer via FFA for each untreated and BFA-treated sample, as previously described for LCMV-eGFP (*n* = 3). Each sample was normalized against untreated 24 hpi titer and averaged between three biological repeats.

### Time-of-addition addition experiments

Trypsinized A549 cells were seeded into 12-well plates at 1 × 10^5^ cells/well and incubated at 37°C. Following 16–24 h, for the 0 hpi time point, the cells were pre-treated with 500 µL BFA at 5,000 ng/mL for 45 min. Subsequently, the cells were infected with rLCMV-eGFP at an MOI of 5 in 500 µL in 10% FBS DMEM media. Of note, for the 0 hpi time point, the 500 µL virus infection media was made in 5,000 ng/mL BFA media. For each time point, 500 µL of BFA at 10,000 ng/mL was added to each well (1:1) to give a final concentration of 5,000 ng/mL. At 12 hpi, for eGFP fluorescence was imaged, cell lysates harvested, and viral supernatants collected.

The eGFP was determined using the Incucyte S3 live-cell imaging software. Total green cell count was recorded, normalized to confluency per well, and then analyzed as a percentage of untreated control. Normalized values were averaged between three biological repeats (*n* = 3). Cells were lysed for analysis via western blotting. Densitometry was performed using Fiji software and NP expression normalized to confluency per well, then analyzed as a percentage of untreated control. Normalized values were averaged between three biological repeats (*n* = 3). Titer was calculated via FFA for each BFA time-of-addition, as previously described for LCMV-eGFP. Each sample was normalized against the untreated control, and averaged between three biological repeats (*n* = 3).

## SUPPLEMENTAL MATERIAL

Data Set S1 jvi.02006-23-s0001.xlsx Data set showing how cellular factors involved in vesicle trafficking impact LCMV-EGFP growth, identified using a curated siRNA library.

Fig. S1 jvi.02006-23-s0002.tiff Colocalization of LCMV components with COPA and AP4E1.

Fig. S2 jvi.02006-23-s0003.tiff Treatment of LCMV-infected cells with BFA inhibits viral processes during multistep growth.

Fig. S3 jvi.02006-23-s0004.tiff Addition of BFA at late stages of rLCMV-GP1-FLAG infection results in changes in the cellular distribution of COPA and AP4E1.

ASM does not own the copyrights to Supplemental Material that may be linked to, or accessed through, an article. The authors have granted ASM a non-exclusive, world-wide license to publish the Supplemental Material files. Please contact the corresponding author directly for reuse.
