# Supplementary figures and images for: Lymphocytic choriomeningitis arenavirus requires cellular COPI and AP-4 complexes for efficient virion production
Source: J Virol. 2024 Feb 9;98(3):e02006-23. doi: 10.1128/jvi.02006-23 (PMC10949467; doi:10.1128/jvi.02006-23)

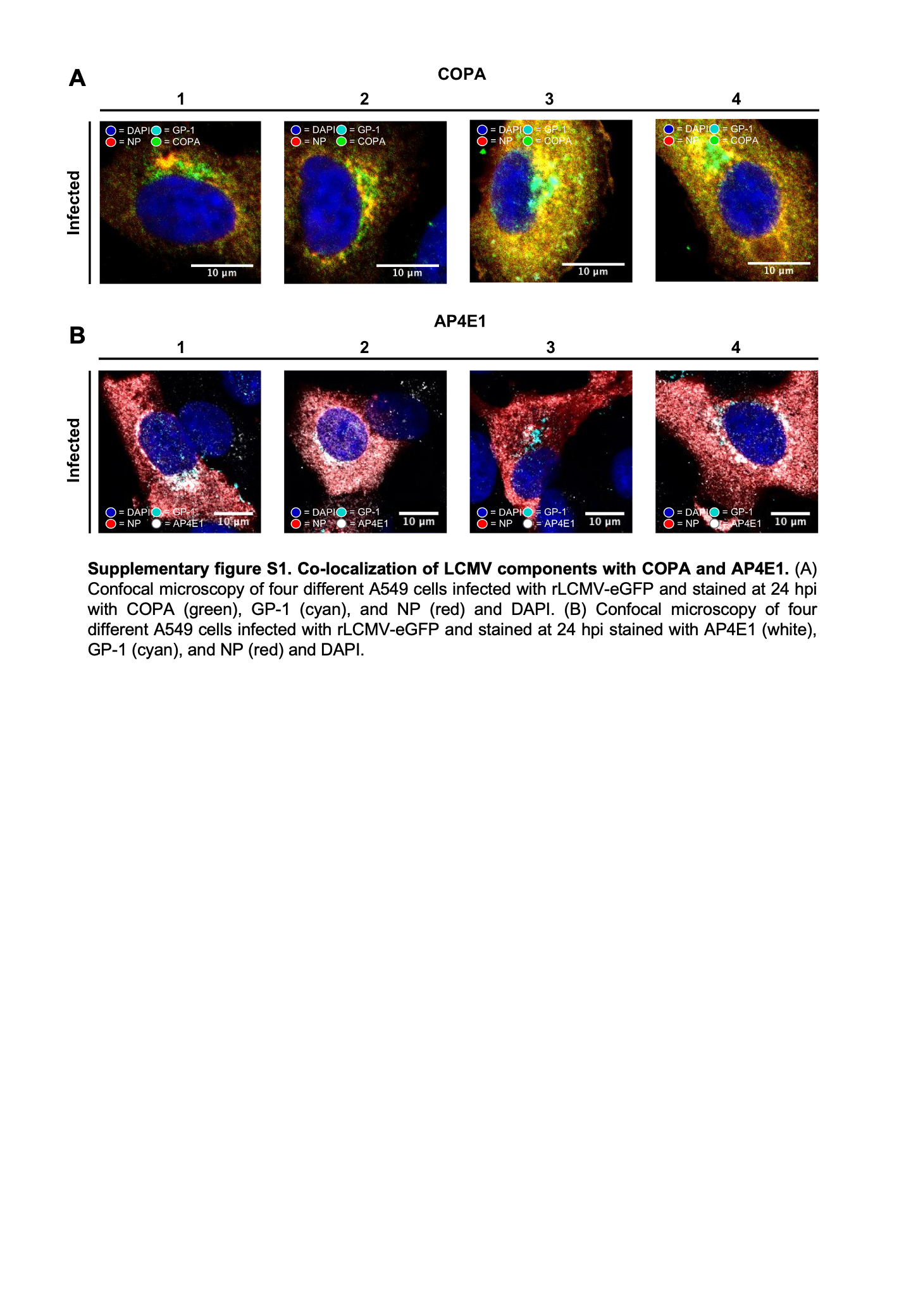

Supplement: Fig. S1 — Colocalization of LCMV components with COPA and AP4E1. [file jvi.02006-23-s0002.tiff]

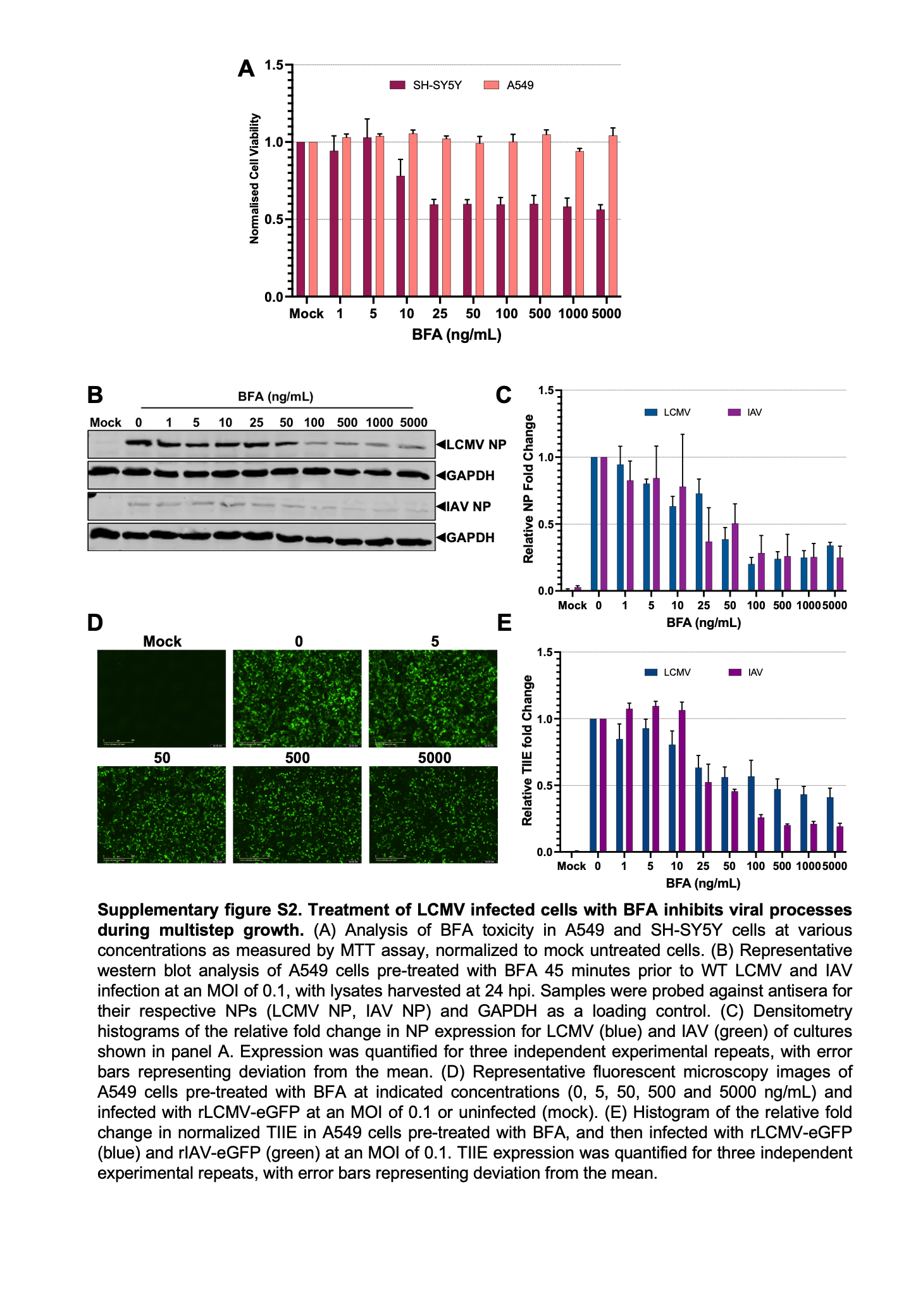

Supplement: Fig. S2 — Treatment of LCMV-infected cells with BFA inhibits viral processes during multistep growth. [file jvi.02006-23-s0003.tiff]

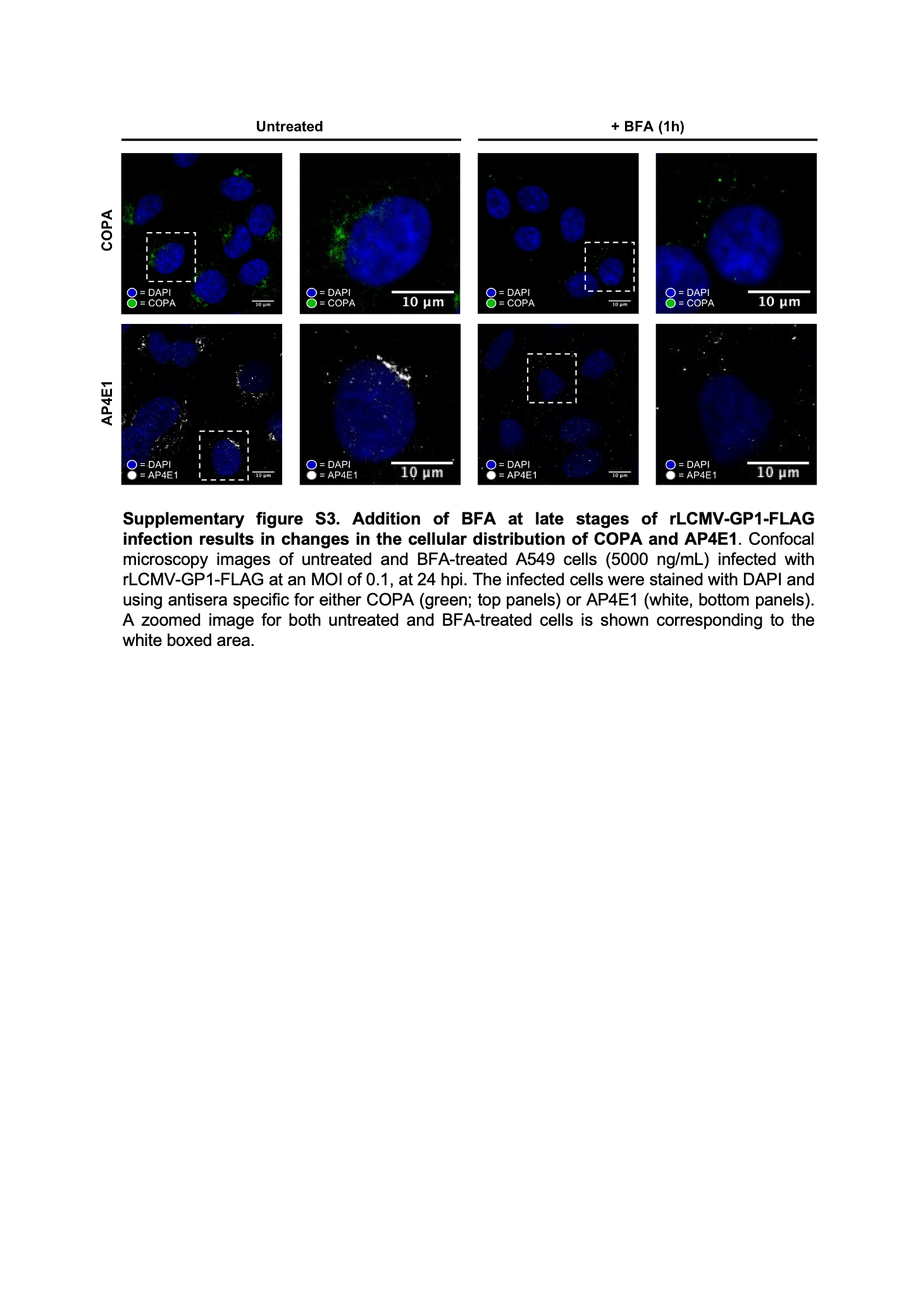

Supplement: Fig. S3 — Addition of BFA at late stages of rLCMV-GP1-FLAG infection results in changes in the cellular distribution of COPA and AP4E1. [file jvi.02006-23-s0004.tiff]
